# Time to shape up – assessment and reporting standards for data quality in clinical research using echocardiographic imaging techniques require improvement

**DOI:** 10.1186/s12872-019-1247-4

**Published:** 2019-12-03

**Authors:** Kai O. Hensel

**Affiliations:** 1 Department of Paediatrics, Cambridge University Hospitals NHS Foundation Trust, Addenbrooke’s Hospital, Cambridge Biomedical Campus, Hills Road, Cambridge, CB2 0QQ UK; 2 Department of Paediatrics, Faculty of Health, Witten/Herdecke University, Center for Clinical & Translational Research (CCTR), Witten, Germany

**Keywords:** Variability, Validity, Strain imaging, Echocardiography, Bias, Quality improvement, Clinical trials, Quality assessment

## Abstract

Advanced echocardiography techniques such as speckle tracking imaging are sensitive diagnostic tools frequently used in various clinical and scientific scenarios. Importantly, imperfect reproducibility and dependence of post-processing algorithms on echocardiographic image quality are potential methodological limitations. Therefore, meticulous assessment of data quality and detailed reporting of study methodology, sample specifics, technical peculiarities and measurement conditions are crucial. Unfortunately, despite the recognized importance of this, there is still no broadly accepted standard for assessing the quality of echocardiographic images in clinical research reports. This article quintessentially highlights important shortcomings of data quality assessment and methodological study design, commonly occurring in clinical research reports using advanced echocardiography techniques. Finally, suggestions are made as to how researchers, scientific communities and biomedical journals can contribute to the ever-lasting process of improving the quality of clinical research in cardiovascular imaging.

## Background

I read with great interest the recent article entitled “Left ventricular short-axis systolic function changes in patients with hypertrophic cardiomyopathy detected by two-dimensional speckle tracking imaging” by Huang et al., published in BMC Cardiovascular Disorders [1]. While the medical topic and chosen methodology (i.e. layer-specific myocardial deformation assessment) are important and the article undoubtedly provides interesting insights, it also elicits the need for an overdue focus on data quality assessment, (non-)standardized research reporting, and benchmarking of biomedical imaging studies in clinical science.

Cardiovascular ultrasound and its quantitative add-on speckle tracking echocardiography are powerful diagnostic tools both in biomedical research and clinical practice. However, echocardiographic validity is limited by real and perceived suboptimal measurement reproducibility [2, 3]. Therefore, caution must be paid when using this sensitive tool. Specifically, rigorous reporting of the research setting, methodological design, study samples, measurement conditions and data quality are key prerequisites to interpret research results in the right light. Unfortunately, hitherto there is still no broadly accepted standard for assessing the quality of echocardiographic measurements, let alone mandatory reporting standards in clinical research studies using biomedical imaging techniques.

## Main text

The study by Huang and colleagues is a useful example of learning points and room for improvement that concern many timely reports using quantitative biomedical imaging such as speckle-tracking echocardiography. Regarding echocardiography-specific study features, the methodological design lacks three important quality assurance measures: echocardiographic image quality reporting, examiner blinding to subjects’ study cohort affiliation, and inter-observer variability testing.

First, we and others have shown the varying influence of echocardiographic image quality for myocardial deformation analyses using speckle tracking echocardiography [4] and other quantitative assessments [5]. Importantly, substandard images may yield “implausibly normal” deformation parameters and are therefore prone to result in misinterpretation of potentially impaired myocardial mechanics. In addition to image quality, other key features such as foreshortening of the views [6, 7], sampling frame rate [8], heart rate [9] and LV morphology [10] may affect the measurements performed by speckle tracking echocardiography. A critical review of current approaches for echocardiographic quality benchmarking in clinical research recently pointed out, that despite its recognized importance, there is still no broadly accepted standard for assessing the quality of echocardiographic measurements in clinical research reports [11]. Nevertheless, researchers should make an effort to overcome this gap, e.g. by documenting at least the number of non-visualized or excluded viewing planes and segments. Moreover, societies and working groups should establish and validate a uniformly-accepted quality scoring system and reporting standards for biomedical imaging, such as the famous CONSORT statement for the reporting of clinical trials [12]. A step in the right direction is the EACVI/ASE/Industry Task Force’s significant efforts to standardize LA, RA and RV deformation imaging [13]. However, their focus was purely clinical, thus of little help to matters of strain imaging data acquisition and reporting in research. A similar endeavor for the scientific use of quantitative echocardiography ought to follow. Once established, biomedical journals should make these standards mandatory for scientific publishing, to increase adherence to these important recommendations.

Second, other than this society-based shortcoming, the importance of blinding in clinical studies has long been established [14]. Therefore, the reason for the lack of examiner blinding to subjects’ study cohort affiliation in Huang’s work remains unclear. The risk of bias is high if adequate blinding is not used, as proven by strong empirical evidence published two decades ago [15]. Hence, researchers, ethics committees and biomedical editors should avoid the design (permission, or publication, respectively) of unblinded clinical studies as appropriate, and continue on an ever-improving – yet often rocky - road to excellence and scientific rigor in clinical research design.

Third, given the known variances in echocardiographic reproducibility, the absence of inter-observer variation testing in Huan’s study is a surprising limitation. Even well-conducted large trials have yielded low interobserver reproducibility (coefficient of variation increasing 70%) and a large proportion of non-assessable echocardiographs (e.g. up to 50% of tissue-Doppler imaging), which makes the study findings hard to interpret [2]. Therefore, the assessment of reproducibility for sensitive diagnostic tools in clinical research should become a *conditio* sine qua non*.*

For the sake of completeness, two non-echocardiography-specific limitations in the study of Huang et al. deserve mention. The amount of statistical comparisons and *p*-value calculations without Bonferroni correction or prospective prioritization constitute a biostatistical weakness. In light of the controversial nature of Bonferroni correction and its newer alternatives [16], reducing the risk of a false negative outcome (the type II error), should be achieved by increasing sample size when multiplicity corrections are performed. Finally, elegantly conducted and widely cited works had delivered conflicting results, as compared to the present study, and therefore deserve to be critically discussed [17, 18].

## Conclusion

As a scientific community, we owe our readership – and our patients – thorough methodological study designs as well as rigorous analyses of both the promising findings as well the potential limitations and, ultimately, the learning opportunities that each study brings to the table. The lack of benchmarking and standardized quality assurance in biomedical imaging is a ubiquitous challenge that ought to be addressed by study designers and scientific societies in the future.

### Acknowledgements

I want to express my gratitude to the voluntary peer-reviewers and the handling editor for their scientific contributions.

### Authors’ contributions

KOH wrote the manuscript.

### Funding

Not applicable.

### Availability of data and materials

Not applicable.

### Ethics approval and consent to participate

Not applicable.

### Consent for publication

Not applicable.

### Competing interests

The author declares that he has no competing interests.
