# Supplementary material for: Time to shape up – assessment and reporting standards for data quality in clinical research using echocardiographic imaging techniques require improvement
Source: BMC Cardiovasc Disord. 2019 Dec 3;19:281. doi: 10.1186/s12872-019-1247-4 (PMC6891971; doi:10.1186/s12872-019-1247-4)
Supplement: Related texts

# Reply: Author’s response

**Authors:** Jun Huang

Thank you very much for your letter. I wish to publish a response to the correspondence “Time to shape up - assessment and reporting standards for data quality in clinical research using echocardiographic imaging techniques require improvement”.

Responses to the article:

Thanks for the critical comments to our group about the article “Left ventricular short-axis systolic function changes in patients with hypertrophic cardiomyopathy detected by two-dimensional speckle tracking imaging” [1]. Dr. Hensel mainly presented three main problems, which include echocardiographic image quality reporting, examiner blinding to subjects’ study cohort affiliation, and inter-observer variability testing.

Firstly, echocardiographic image quality reporting, in our study [1], all echocardiographic images or videos were acquired by an experienced chief physician who was a specialist in echocardiography. All of these images or videos were clear and could be analyzed by EchoPAC software. If hypertrophic cardiomyopathic patients or normal control subjects presented poor echocardiographic images or videos, we excluded these patients from the analysis.

Secondly, examiner blinding to subjects’ study cohort affiliation is really important to our research. However, in this this study [1], we enrolled hypertrophic cardiomyopathic patients and normal subjects for detecting left ventricular (dys)-function. The experimental design was “blinded”, although an experienced echocardiography consulant, could diagnosis the hypertrophic cardiomyopathic patients form the images and videos quickly.

Thirdly, in this study [1], we ignored the inter-observer variability testing. All the results were measured by the author of this correspondence. Inter-observer and intra- observer variability testing are very important for the reliability in the clinical research. In our future research, we have included the inter- observer and intra- observer variability testing in our manuscript.

Thanks to Dr. Hensel, I and my group members agree with the recommendation about the standard for the quality of echocardiographic measurements, “blinding” analysis and variability testing, and these elements are very crucial for ongoing and future clinical research.

Finally, I have a suggestion, Dr. Hensel should make a “model” example in a future piece, because most publications have the same issues, and he help propose the standard for future research. Thank for the critical advice once again.
